# Systemic Immune-Inflammation Index May Predict Mortality in Neuroblastoma

**DOI:** 10.7759/cureus.35705

**Published:** 2023-03-02

**Authors:** Ilknur Banlı Cesur, Zerrin Özçelik

**Affiliations:** 1 Pediatric Surgery, Adana City Training Hospital, Adana, TUR

**Keywords:** neutrophil-to-lymphocyte ratio, systemic immune-inflammation index, inflammation, mortality, neuroblastoma

## Abstract

Introduction: Neuroblastomas (NB) are among the most frequent childhood solid tumors. The link between inflammation and cancer is well understood. Many research studies have been conducted to determine the prognostic importance of inflammatory markers in cancer patients. C-reactive protein (CRP), neutrophil-to-lymphocyte ratio (NLR), platelet-to-lymphocyte ratio (PLR), and systemic immune-inflammation index (SII) are all potential inflammation indicators. The purpose of this study is to assess the efficacy of NLR and SII as inflammatory indicators in predicting NB patient survival.

Materials and methods: Patients with NB diagnosed between January 1, 2012 and December 31, 2021 were studied retrospectively, and death was documented. By dividing the number of neutrophils by the number of lymphocytes, the NLR was obtained. The SII was calculated by multiplying the NLR by the platelet count.

Results: 46 patients with NB were included in the study with a mean age of 57.58 months (4.14-170.05). When the patients were analyzed based on mortality the NLR and SII values were statistically significantly increased in the dead group (2.71 (1.22-4.1 ) vs. 1.7 (0.16-5.1); p=0.02; and 677.8 (215-1322) vs. 294.6 (69.49-799.1), respectively; p=0.012). Analysis of the receiver operating curve found that 328.49 is the ideal cutoff value for SII to predict mortality with a sensitivity of 83% and a specificity of 68% (area under the receiver operating characteristic curve = 0.814 (95% confidence interval: 0.671-0.956), p=0.005 ). Analyzing the influence of risk factors on survival using Cox regression analysis, SII was discovered as a significant predictor of survival in the study (HR =1.001, 95% CI =1-1.20; p=0.049).

Conclusion: SII may be used to predict the overall survival of NB patients.

## Introduction

Studies on the role of inflammation in cancer genesis have been conducted [1]. Inflammation has been related to cancer growth and treatment resistance by increasing proliferation and angiogenesis while inhibiting apoptosis. Anti-inflammatory drugs are also being studied for their potential use in tumor prevention and treatment [2,3].

Blood inflammation biomarkers, or inflammation-based scores, have been used as cancer survival predictors in recent years. The systemic immune-inflammation index (SII), C-reactive protein (CRP), and neutrophil-to-lymphocyte ratio (NLR) are among the most often used inflammatory markers [4,5].

Neuroblastoma (NB) is the most prevalent solid extracranial pediatric malignancy [6,7]. Adult ganglioneuroma (GN), less mature ganglioneuroblastoma (GNB), and immature NB are all types of NBs [6,8]. Despite the fact that the overall survival (OS) rate for NB has grown in recent decades, the 5-year OS rate for all children diagnosed with NB remains at 71% [6]. Studies have found a relationship between NB and inflammatory markers [8,9].

The goal of this study was to see how NLR and SII, two markers of inflammation, predicted survival in NB patients.

## Materials and methods

Patients with NB who were admitted to the hospital between January 1, 2012 and December 31, 2021 were assessed retrospectively. Patients having a histological diagnosis of NB and enough follow-up data were included in the research. This study finally included 46 children who had complete data. Following the initial therapy, patients were monitored every three months for the first three years, then every six months thereafter. Clinical information such as the patient’s age at diagnosis, gender, International Neuroblastoma Staging System (INSS) stage, metastatic status at diagnosis, and tumor size was gathered.

This probe was sanctioned by the hospital’s ethics committee. The study followed the Helsinki Declaration’s ethical requirements. Legal guardians for all participants provided written informed permission. This study was approved by Adana Şehir Eğitim ve Araştırma Hastanesi, Klinik Araştırmalar Etik Kurulu (Approval number: 2196).

Complete blood counts were obtained from patient files prior to therapy. NLR was calculated by dividing the number of neutrophils by the number of lymphocytes. By dividing the NLR by the platelet count, the SII was computed.

SPSS 25.0 for Windows (Statistical Package for the Social Sciences, Chicago, IL) was used for statistical analysis. Unless otherwise indicated, continuous variable data is normally reported as the mean and standard deviation. To compare groups, the student’s t-test (for normal distribution data) or the Mann-Whitney U test (for non-normal distribution data) were employed. We compared categorical variables with the Chi-square test. The effects of many determinants on mortality were examined using univariate analysis. In univariate analysis, the model’s parameters obtained a p-value of 0.05. The cutoff value for SII and NLR for predicting mortality formation was obtained using a receiver operating characteristic (ROC) curve analysis. The best cutoff value was chosen based on the ROC analysis’s maximum sensitivity and specificity values, p=0.05 was seen as crucial on both sides.

Survival curves were created using the Kaplan-Meier method and evaluated using the Log-rank test. All two-tailed tests required a p-value of 0.05 to be statistically significant.

## Results

The current study comprised 46 patients with a histological diagnosis of NB, 21 (45.7%) of whom were female. The average age was 57.58 months (4.14-170.010). Abdominal mass was the most frequent presenting symptom (n = 32, 69.6%). On ultrasonography, 24 patients had masses less than 5 cm; however, this number was reduced to 21 patients (45.7%) on CT. Undifferentiated or poorly differentiated tumors accounted for 18 (38.1%) of the malignancies, whereas unknown tumors accounted for 20 (43.5%). A severe sickness plagued 39 (84.6%) of the hospitalized patients. Despite the fact that 29 (63%) of the patients had metastases, only 11 (23.9%) had lymph node involvement. The majority of the patients (78.3%) had already received chemotherapy. SII and NLR average values were 394.57 (69.49-1322) and 1.96 (0.0-5.1), respectively. The average duration of follow-up was 33.74 (2.76-107.41) months, with 12 patients (26.1%) dying beyond the term of observation (Table 1).

**Table 1:** General properties of the patients NLR: neutrophil to lymphocyte ratio; SII: systemic immune-inflammation index

	N=46 (%)		
Gender (Male)	25 (54.4%)		
Age (months)	57.5 (4.14-169.05)		
Complaint	Abdominal mass		32 (69.6%)
	Fever		2 (4.4%)
	Antenatal		2 (4.4%)
	Bone pain		1 (2.2%)
	Thoracal mass		1 (2.2%)
	Neurologic		1 (2.2%)
	Vision loss		1 (2.2%)
	Other		6 (12.5%)
Diagnosis	Biopsy		16 (34.8%)
	Clinic-radiologic-bone marrow aspiration		30 (65.2%)
Ultrasonography	≤5 cm		24 (52.2%)
	>5 cm		22 (47.8%)
CT	≤5 cm		21 (45.7%)
	>5 cm		25 (54.3%)
PET	SUV_max _≤2.6		15 (65.2%)
	SUV_max_ >2.6		8 (34.8%)
Histopathology	Differentiated		5 (10.9%)
	Undifferentiated + poorly differentiated		18 (39.1%)
	Unknown		20 (43.5%)
	Intermixed		3 (6.6%)
Stage	Early (1,2)	7 (15.2%)	
	Advanced (3,4)	39 (84.8%)	
Metastasis	Present	29 (63%)	
	Absent	17 (37.7%)	
Lymph node involvement	Present	11 (23.9%)	
	Absent	35 (76.1%)	
İnvolvement site	Right	24 (52.2%)	
	Left	19 (41.3%)	
	Bilateral	1 (2.2%)	
	Paravertebral	2 (4.3%)	
Neoadjuvant chemotherapy	Present	36 (78.3%)	
	Absent	10 (21.7%)	
Lymphocyte number	4390 (940-7630)		
Neutrophil number	4281 (1170-8430)		
Platelet number	343.43 (22-890)		
NLR	1.96 (0.16-5.1)		
SII	394.57 (69.49-1322)		
Ferritin (ng/ml)	225.3 (0.4-1322)		
Follow-up (months)	33.74 (2.76-107.41)		
Mortality	12 (26.1%)		

The patients were divided into two groups based on their mortality (Group 1 = died, Group 2 = alive). Age, gender, and tumor mass as measured by USG or CT were not substantially different across groups (all p > 0.05) (Table 2). Only the NLR and SII values differed between the two groups significantly (2.71 (1.22-4.1) vs. 1.7 (0.16-5.1); p=0.02; and 677.8 (215-1322) vs. 294.6 (69.49-799.1)), respectively; p=0.012.

**Table 2:** Comparison of the patients who died and who are alive NLR: neutrophil to lymphocyte ratio; SII: systemic immune-inflammation index

	Group 1 (Dead, N=12)		Group 2 (Alive, N=34)	p-value
Gender (Male)	7 (58.8%)		18 (52.9%)	0.747
Age (months)	71.74 (22.16-169.05)		52.48 (4.1-134.1)	0.140
Ultrasonography (≤5 cm)	7 (58.3 %)		17 (50%)	0.619
CT (≤5 cm)	5 (41.7%)		16 (47.1%)	0.747
PET (SUV max≤2.6)	3 (12%)		12 (75%)	0.136
Histopathology	differentiated	-	5 (14.7%)	0.201
	Undifferentiated + poorly differentiated	3 (25%)	15 (44%)	
	Unknown	8 (66.7%)	12 (35.3%)	
	Intermixed	1 (8.3%)	2 (5.8%)	
Stage (Early 1,2)	0 (0%)		7 (20.6%)	0.708
Metastasis (Present)	9 (75%)		20 (58.8%)	0.318
Lymph node involvement (Present)	3 (25%)		8 (23.5%)	0.667
Neoadjuvant chemotherapy (Present)	12 (100%)		24 (70.6%)	0.034
Lymphocyte number	4027 (1880-6170)		4518 (940-7630)	0.444
Neutrophil number	4428 (2520-6380)		4229 (1170-8430)	0.294
Platelet number	297.33 (22-890)		359.1 (33-664)	0.406
NLR	2.71 (1.22-4.1)		1.7 (0.16-5.1)	0.02
SII	677.8 (215-1322)		294.6 (69.49-799.1)	0.012
Ferritin (ng/ml)	249 (91.6-384)		219.6 (9-787)	0.412

With a sensitivity of 83% and a specificity of 68%, the ROC curve revealed that 328.49 is the optimal cutoff value for SII to predict mortality (area under the ROC curve = 0.814 (95% confidence interval: 0.671-0.956), p=0.005).

**Figure 1:**
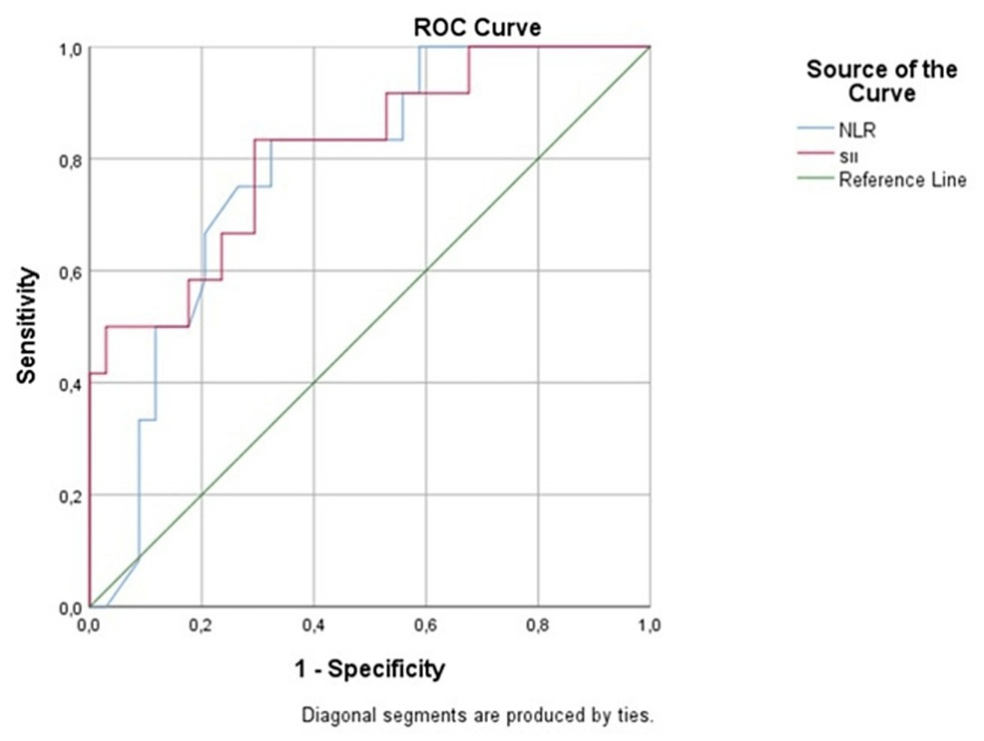
ROC analysis of NLR and SII NLR: neutrophil to lymphocyte ratio; ROC: receiver operating curve; SII: systemic immune-inflammation index

The ideal NLR cutoff value of 1.63 effectively predicted mortality with an 82% sensitivity, 65% specificity, and 0.778 area under the curve (AUC) (p=0.005; confidence interval: 0.638-0.918) (Figure 1).

SII was revealed as a significant predictor of survival in a survival study (HR = 1.001, 95% CI = 1-1.20; p = 0.049) using Cox regression analysis to analyze the influence of risk factors on survival. According to Cox regression analysis, gender, distant metastasis, NLR, tumor mass evaluated by USG or CT, stage, lymph node involvement, and age had no influence on death.

A Kaplan-Meier survival analysis was done using the cutoff values obtained from the ROC analysis. Patients with increased SII levels showed a worse OS rate than those with normal SII levels ((46.87 (30.5-63.24) vs. 97.53 (84.52-110.5); p=0.001); ((45.278 (29.6-61.01) vs. 94.647 (78.37-110.9); p= 0.001). Patients with a high NLR level had significantly worse OS than those with a median NLR level.

## Discussion

Inflammation enhances the risk of cancer and may influence its stage and development [2,10]. Lymphocytes in tumors can be used to classify cancer [11]. Increased immune cell infiltration and systemic inflammation may potentially contribute to cancer growth [12,13].

A substantial amount of study has been conducted on the association between NB and inflammation [9]. Chemokine receptor-4 (CXCR4) expression was shown to be much higher in advanced NB tumors, and these tumors had a worse prognosis than other forms of NB [9]. Sufficient amounts of the pro-inflammatory transcription factor NF-B are required for the survival of S-type NB tumor cells. IL-6 has the capacity to increase the proliferation of NB cells that express IL-6-R. These cells are resistant to etoposide-induced apoptosis, and higher levels of IL-6 were observed in the blood of NB patients with bone metastases [9].

Furthermore, there is evidence that interactions between NB tumor cells and inflammatory cells contribute to NB growth and dissemination [14]. The number of tumor-associated macrophages, or TAM, was shown to be increased in metastatic NB [15]. It is likely that bone marrow-derived mesenchymal stem cells (BM-MSCs) and peripheral blood mononuclear cell-derived macrophages will be attracted to the tumor site and activated into cancer-associated fibroblasts and TAMs (CAFs). These changes create an environment that is conducive to the development of NB [16,17].

The SII was shown to be substantially linked with the OS of NB patients in both univariate and multivariate survival studies that controlled for clinical risk variables. The ROC curve and AUC analysis revealed that SII is a predictor of NB patients’ OS.

There are several types of malignancies, and the SII index has been demonstrated to have significant connections with cancer prognosis [15-18]. A high SII indicates an increase in platelets and/or neutrophils while decreasing the number of lymphocytes. Neutrophils are thought to have a considerable effect on the formation of cancer, its progression, and its propensity to spread [19]. A number of ligands can attract neutrophils, and neutrophils’ production of matrix metalloproteinase-9, reactive oxygen species, and reactive nitrogen species may play a role in cancer growth [20]. Another method that can stimulate angiogenesis is the production of vascular endothelial growth factors by neutrophils [20]. Neutrophils are another type of cell that can contribute to cancer growth. They accomplish this by impairing the capacity of natural killer cells to remove cancer cells and boosting cancer cell motility [20].

According to a recent study, platelets create bioactive chemicals such as lipids, microRNAs, and growth factors that play critical roles in carcinogenesis, such as angiogenesis and metastasis [21,22]. Platelets and growth factors can both be used to predict chemotherapy response. For people with breast cancer who have a low platelet count or PLR, neoadjuvant therapy is more likely to be completely successful [23].

A reduction in peripheral lymphocytes can impair anti-cancer immunity and cancer immune surveillance [24,25]. SII is calculated by counting neutrophils, lymphocytes, and platelets. SII is thought to be more sensitive than other indicators in diagnosing inflammation-related mortality.

There are several drawbacks to this study. Selection bias is possible because this was retrospective research. Second, because this was a single-center study with limited sample size, it is difficult to extrapolate the findings to the entire community. Third, due to technological difficulties, we were unable to determine the MYCN status of the patients.

## Conclusions

The SII was found to predict OS in individuals with NB in this retrospective analysis. Prospective studies with bigger sample numbers are required to validate the findings of this study.
